# Impact of socioeconomic status and remoteness of residence on pregnancy outcome in major congenital heart disease: mediation analysis

**DOI:** 10.1002/uog.70145

**Published:** 2026-01-03

**Authors:** S. Bennett, L. K. Hornberger, A. Kaur, D. Fruitman, L. G. Eckersley

**Affiliations:** ^1^ Division of Cardiology, Department of Pediatrics University of Alberta Edmonton AB Canada; ^2^ Fetal & Neonatal Cardiology Program, Division of Cardiology, Department of Pediatrics, Women & Children's Health Research Institute University of Alberta Edmonton AB Canada; ^3^ Division of Cardiology, Department of Pediatrics University of Calgary, Alberta Children's Hospital Calgary AB Canada

**Keywords:** cardiac, echocardiography, fetus, mediation, remoteness, SES

## Abstract

**Objective:**

Increased remoteness of residence (RoR) and lower socioeconomic status (SES) negatively impact the rate and gestational age (GA) at the diagnosis of major congenital heart disease (mCHD). We examined the direct and indirect relationships of RoR from a tertiary fetal cardiology center and Chan SES index with the rate of termination of pregnancy (TOP).

**Methods:**

We conducted a retrospective population‐based cohort study of all pregnancies with a prenatal diagnosis of mCHD in Alberta, Canada, from January 2008 to December 2021. Maternal RoR from the nearest tertiary fetal cardiology center and Chan SES index were primary exposures and GA at diagnosis of mCHD was examined as a potential mediator. The outcome was TOP. RoR, SES and GA at diagnosis were analyzed using structural equation modeling and mediation analysis, adjusting for maternal age, parity and presence of syndromic diagnosis.

**Results:**

Of 1091 included pregnancies with mCHD and known pregnancy outcome, 203 (18.6%) ended in TOP. A lower rate of TOP was associated with diagnosis ≥ 22 weeks' gestation (47/532 (8.8%)) compared with diagnosis < 22 weeks (156/559 (27.9%)) (*P* < 0.001). There was a significant reduction in the rate of TOP among those with RoR ≥ 100 km (relative risk (RR), 0.94 (95% CI, 0.89–0.99); *P* = 0.022), and a trend towards an association between higher SES and increased likelihood of TOP (RR, 1.11 (95% CI, 1.00–1.22); *P* = 0.053), mediated by GA at diagnosis. There was no direct effect of RoR or SES on the rate of TOP. Diagnosis ≥ 22 weeks was associated with younger nulliparous (*P* = 0.018) and parous (*P* = 0.036) mothers, but not with maternal age overall. When stratified by the presence of a comorbid syndromic diagnosis, lower rates of TOP were indirectly associated with greater RoR and lower SES in fetal mCHD with syndromic diagnosis, mediated by GA at diagnosis (*P* = 0.057 and *P* = 0.02 respectively).

**Conclusions:**

The parental decision to terminate pregnancy was related directly to GA at diagnosis of mCHD and related indirectly to greater RoR when mediated by GA at diagnosis. These findings should prompt further exploration of factors responsible for the later diagnosis of mCHD in those residing remotely. © 2026 The Author(s). *Ultrasound in Obstetrics & Gynecology* published by John Wiley & Sons Ltd on behalf of International Society of Ultrasound in Obstetrics and Gynecology.

## INTRODUCTION

Congenital heart disease (CHD) is a significant health issue affecting 12.3 per 1000 live births, with approximately 10–20% of cases requiring invasive intervention within the first year after birth^1^, ^2^, ^3^. In Canada, CHD affects approximately 1 in 100 live births, with surgical or catheter‐based intervention performed in around 2–3 per 1000 live births annually^4^. Maternal geographic location of residence and socioeconomic factors influence both the incidence and outcomes associated with major CHD (mCHD)^5^, ^6^, ^7^, ^8^, ^9^, ^10^. Increased remoteness of residence (RoR) from centralized services and lower socioeconomic status (SES) have also been shown to negatively impact prenatal detection rates of mCHD and gestational age (GA) at diagnosis, with findings varying by health jurisdiction^11^, ^12^, ^13^, ^14^, ^15^.

The population‐based cohort study of Kaur *et al*.^11^ reported that fetuses of women residing in rural areas of the Canadian province of Alberta, at a greater distance from regional fetal cardiology centers, were diagnosed with mCHD at a significantly higher GA than those living in urban areas. These findings and those of similar studies suggest that physical determinants of health, specifically geographic barriers, may limit access to obstetric screening and specialized diagnostic resources, contributing to delays in the diagnosis of mCHD^16^, ^17^, ^18^, ^19^.

Although prior studies have explored the association between SES, RoR and access to prenatal care, there are limited data on how these factors influence decision‐making in pregnancy following a prenatal diagnosis of mCHD. Additionally, there is a gap in the understanding of the extent to which indirect pathways, such as timing of diagnosis, may mediate such relationships.

In the present study, we sought to examine the relationship of RoR, SES and GA at diagnosis of mCHD with the parental choice of termination of pregnancy (TOP). We hypothesized that lower SES and increased RoR would be associated with later GA at diagnosis of mCHD, and that later GA at diagnosis would, in turn, be associated with a lower rate of TOP.

## METHODS

### Study population

This retrospective study included all pregnancies with a prenatal diagnosis of mCHD (defined as a significant congenital structural cardiac defect requiring or expected to require surgical intervention within the first year after birth), diagnosed in Alberta, Canada, from January 2008 to December 2021. Cases were identified based on a diagnosis of CHD and the timing of surgical procedures performed, with review of fetal echocardiography reports to confirm mCHD where necessary. Cases with unknown pregnancy outcome were excluded from mediation analysis. Those undergoing catheter intervention only were also excluded owing to inadequate data availability for the primary outcome and mediation analysis.

### Data collection

Data collection involved review of the fetal cardiology database, healthcare facility records, surgical referral records and fetal cardiology reports. Information collected included maternal age, parity, fetal mCHD diagnosis, GA at fetal echocardiographic diagnosis of mCHD, syndromic diagnosis and pregnancy outcome (TOP or intention to continue the pregnancy (live birth or intrauterine fetal demise (IUFD))).

To assess the impact of mCHD severity, cases were classified as single‐ventricle (SV) physiology (hypoplastic left heart syndrome, tricuspid atresia, unbalanced atrioventricular septal defect or other complex SV lesion) or biventricular (BV) physiology (all other cases).

RoR was determined based on the direct distance (‘as the bird flies’ approach) from the six‐digit postal code of the maternal residence to the nearest fetal cardiology unit, calculated using geocoding. In Alberta, Canada, there are two fetal cardiology units, one based in Edmonton and one in Calgary. As the majority of the population of Alberta is concentrated in high‐density regions around these two metropolitan cities, with rural and remote low‐density regions existing ≥ 100 km from these cities, RoR was analyzed both categorically, as < 100 km or ≥ 100 km from the closest tertiary fetal cardiology center, for descriptive comparisons, and continuously, as distance in 100‐km increments, for mediation and regression analysis.

Maternal residential postal code was used to assign the Chan SES index^20^, a validated census‐based Canadian neighborhood‐level SES index based on dissemination areas (of 400–700 people) that incorporates 22 variables to assess material and social deprivation. Material deprivation factors include income, education and employment, while social deprivation indicators include living alone, marital status and family structure. The Chan SES index integrates additional variables related to cultural identity, environmental pollutants and environmental injustice, providing a more comprehensive measure of SES in Canada^20^. Although data on religious beliefs and cultural backgrounds were not directly available, the Chan SES index was used owing to its ‘cultural identity’ component. The mediation analysis was conducted under the assumption that the Chan SES index partially captures these unmeasured factors. The Chan SES index was utilized both as a normalized scale between –1 and 1, and as quintiles of the Alberta population.

### Outcome measures

Pregnancy outcome was categorized as TOP or intention to continue the pregnancy. GA at the fetal echocardiographic diagnosis of mCHD was recorded. GA at diagnosis was considered an endogenous outcome, and a potential mediator of the association of increased RoR and lower SES with pregnancy outcome. In descriptive analyses, GA at diagnosis was categorized as < 22 weeks or ≥ 22 weeks to reflect clinical relevance, the local viability threshold and ethical considerations surrounding GA and TOP. Locally, in Edmonton, additional ethical committee review is required for consideration of TOP > 23 + 6 weeks' gestation, and it is offered only for severe pathology and late referrals. GA at diagnosis was analyzed as a continuous variable for the mediation analysis. Categorization of diagnosis < 23 weeks and < 24 weeks was also performed in sensitivity analyses.

### Statistical analysis

The paired *t*‐test and Pearson's chi‐square test were used to investigate the difference between groups in maternal age categories (< 35 years, 35–39 years, 40–44 years and ≥ 45 years^21^, and also < 35 years *vs* ≥ 35 years) and parity (nulliparous *vs* parous). RoR was analyzed both as a categorical variable (< 100 km *vs* ≥ 100 km) for descriptive analyses, and as a continuous variable, in 100‐km increments, in regression and mediation models. The Chan SES index quintile was analyzed as a categorical variable for descriptive comparisons and as a continuous scale in mediation models.

Generalized structural equation modeling (GSEM) was utilized to perform mediation analysis and estimate the direct, indirect and total effects of RoR and SES on pregnancy outcome. Interpretations of indirect effects were modeled on the risk ratio scale using Poisson regression and were as follows: negative values (relative risk (RR) < 1) indicate that the predictor decreases the likelihood of TOP through the mediator, while positive values (RR > 1) indicate that the predictor increases the likelihood of TOP. The ratio of indirect‐to‐total effect (RIT) and ratio of indirect‐to‐direct effect (RID) provide additional insight into the strength of the mediation pathways. Maternal age, parity and syndromic diagnosis were considered as confounders because of potential associations with both predictors and outcomes. In our analysis, syndromic diagnosis in particular was associated with both GA at diagnosis (the mediator) and likelihood of TOP, and thus served as a confounder of the mediator–outcome pathway.

The GSEM package in STATA version 14.2 (StataCorp., College Station, TX, USA) was utilized for modeling, with sensitivity analyses including models with and without maternal age, parity and syndromic diagnoses to test model fit. Maternal age and parity were excluded from the final models, as their inclusion reduced model fit and did not materially change the estimates. Stratified analyses were conducted based on the presence of syndromic diagnoses and presence of SV physiology. Logistic modeling of outcome measure can yield inaccurate effect estimates with common outcomes (> 10%) in mediation analysis. An alternative (log‐binomial regression) is unavailable in the statistical package and therefore Poisson regression with robust standard error estimation was used to calculate RR to enhance interpretability. Logit models were also calculated for comparison. The predictor–mediator relationship was estimated using linear regression. Indirect effects were calculated using the product of the beta coefficient method, with total effects equal to the sum of direct and indirect effects. The code used is available on request.

To further explore alternative methodology for mediation analysis, causal mediation using the mediate package in STATA was used to estimate the natural direct effect and natural indirect effect (NIE) of RoR category and Chan SES index quintile. Statistical analysis was conducted using Stata software version 14.2 (StataCorp.). Statistical significance was set at *P* < 0.05.

## RESULTS

### Study Cohort

**Figure 1:**
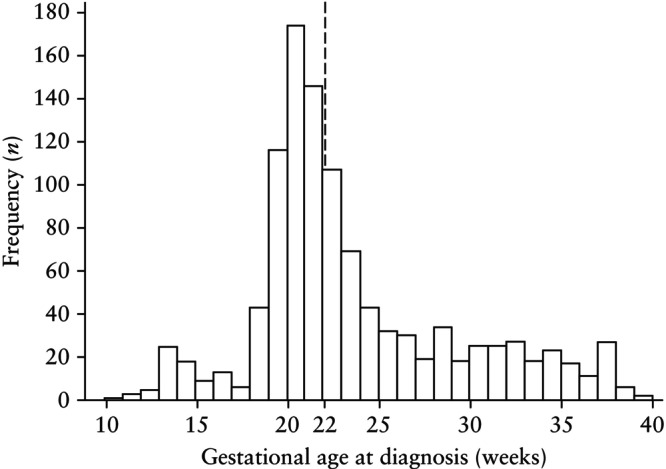
Distribution of gestational age at diagnosis of major congenital heart disease. Dashed line at 22 weeks' gestation separates those with a ‘timely diagnosis’ and those with a late diagnosis in the distribution curve.

From 2008 to 2021, 1098 pregnancies had a prenatal diagnosis of mCHD in Alberta, Canada, with known pregnancy outcome recorded for 1091 cases. Characteristics of the study population are given in Table 1. Mean GA at the time of mCHD diagnosis was 23 + 2 (95% CI, 22 + 6 to 23 + 4) weeks. Of the 1091 pregnancies with known outcome, 559 (51.2%) were diagnosed < 22 weeks and 532 (48.8%) were diagnosed ≥ 22 weeks (Figure 1). The mean and median GAs at diagnosis were 23 + 2 weeks and 21 + 4 weeks, respectively. Prior to 18 weeks there were relatively few diagnoses of mCHD. The distribution of GA at diagnosis is positively skewed and has a positive kurtosis value of 11.7 (Figure 1), indicating more values at the extremes and a high degree of peakedness compared with a normal distribution, which is expected owing to the nature of prenatal detection and screening of mCHD. Of those with a known pregnancy outcome, there were 268 (24.6%) pregnancies with a syndromic diagnosis coinciding with mCHD, 281 (25.8%) cases with SV physiology and 810 (74.2%) cases with BV physiology. The cohort consisted of both singleton (85.2%) and multiple (14.8%) pregnancies. The mean maternal age across the cohort was 30.7 (95% CI, 30.5–31.4) years.

**Table 1:** Characteristics of study population of 1098 pregnancies with prenatal diagnosis of major congenital heart disease, according to whether pregnancy was continued and time of diagnosis < 22 weeks

Characteristic	Continuation of pregnancy*	*P*	Diagnosis < 22 weeks	*P*
Total	888/1091 (81.4)		564/1098 (51.4)	
RoR		0.76		< 0.001
< 100 km	671/835 (80.4)		469/841 (55.8)	
≥ 100 km	217/256 (84.8)		95/257 (37.0)	
Chan SES index quintile†		0.17		0.002
1	191/221 (86.4)		103/222 (46.4)	
2	167/204 (81.9)		88/206 (42.7)	
3	175/220 (79.5)		126/222 (56.8)	
4	161/197 (81.7)		98/197 (49.7)	
5	149/193 (77.2)		116/194 (59.8)	
Maternal age		0.66		0.10
< 35 years	669/818 (81.8)		418/823 (50.8)	
35–39 years	156/198 (78.8)		114/200 (57.0)	
40–44 years	54/65 (83.1)		26/65 (40.0)	
≥ 45 years	9/10 (90.0)		6/10 (60.0)	
Parity‡		0.55		0.25
0	353/438 (80.6)		235/440 (53.4)	
≥ 1	534/651 (82.0)		327/656 (49.8)	
Syndromic diagnosis		0.27		0.74
No	676/823 (82.1)		424/823 (51.5)	
Yes	212/268 (79.1)		140/268 (52.2)	

Data are given as *n*/*N* (%).

* Pregnancy outcome was known for 1091/1098 pregnancies.

† Data missing for 56 patients for continuation of pregnancy and 57 patients for diagnosis < 22 weeks.

‡ Data missing for two patients for continuation of pregnancy and two patients for diagnosis < 22 weeks. RoR, remoteness of residence; SES, socioeconomic status.

### Pregnancy outcome

**Figure 2:**
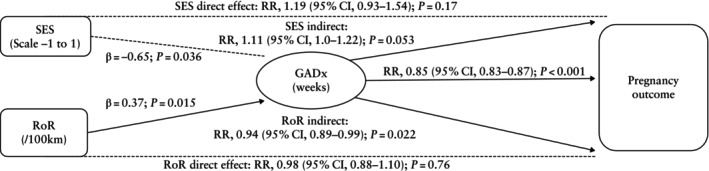
Mediation modeling analysis showing relationship of socioeconomic status (SES) and remoteness of residence (RoR) with pregnancy outcome in cases with prenatal diagnosis of major congenital heart disease, directly and when mediated by gestational age at diagnosis. Neighborhood SES, measured by Chan SES index scale, and RoR, measured in 100‐km increments from closest tertiary fetal cardiology center, are seen to have no direct association with pregnancy outcome (termination of pregnancy (TOP)). However, lower SES and increased RoR are associated with later gestational age at diagnosis (GADx) and, indirectly, with lower rate of TOP. Beta is the coefficient in linear model in each case. RR, relative risk.

Overall, 203 pregnancies (18.6%) ended in TOP, with 156 (76.8%) of all TOP seen in cases with a diagnosis of mCHD < 22 weeks, 178 (87.7%) cases diagnosed < 23 weeks and 191 (94.1%) diagnosed < 24 weeks. TOP was carried out in 47 (8.8%) cases diagnosed ≥ 22 weeks, 25 (5.9%) cases diagnosed ≥ 23 weeks and 12 (3.4%) cases diagnosed ≥ 24 weeks. Later GA at diagnosis (≥ 22 weeks) was strongly associated with a lower rate of TOP (*P* < 0.001) (Figure 2). On univariable analysis, RoR category (analyzed categorically as < 100 km *vs* ≥ 100 km) and Chan SES index (analyzed by quintile) were not directly associated with TOP (*P* = 0.76 and *P* = 0.17, respectively) (Table 1). The rate of TOP did not vary according to maternal age, parity or comorbid syndromic diagnosis. TOP was significantly more frequent in SV physiology cases compared with BV physiology cases (29.9% *vs* 14.8%; *P* < 0.001). On multivariable analysis, SV physiology was a significant predictor of TOP (odds ratio (OR), 2.4 (95% CI, 1.7–3.3); *P* < 0.001), after adjusting for SES, RoR and GA at diagnosis.

### Gestational age at diagnosis

On univariate analysis, increased RoR (≥ 100 km) and lower Chan SES index quintile were significantly associated with diagnosis ≥ 22 weeks (*P* < 0.001 and *P* = 0.002, respectively) (Table 1). The rate of diagnosis < 22 weeks did not vary overall according to maternal age stratification. Analysis using GA < 23 weeks at diagnosis as a cut‐off point yielded qualitatively similar results, whereas using GA < 24 weeks at diagnosis as a cut‐off point led to unbalanced groups and less sensitivity to detect differences in early or late diagnosis among SES quintiles (Table S1). GA at diagnosis varied in subgroups, with diagnosis ≥ 22 weeks occurring more frequently in younger nulliparous mothers (*P* = 0.018) and younger parous mothers (*P* = 0.036) (Table S2). No association was found of mCHD diagnosis < 22 weeks with comorbid syndromic diagnosis (*P* = 0.74) (Table 1) or with maternal age in the presence of comorbid syndromic diagnosis (*P* = 0.072) (Table S2). Cases with SV physiology were more likely to be diagnosed < 22 weeks than were those with BV physiology (57% *vs* 49%; *P* = 0.012). Later GA at diagnosis (≥ 22 weeks) was associated with a significantly lower likelihood of TOP in both the SV and BV physiology groups (SV: OR, 0.29 (95% CI, 0.16–0.52), *P* < 0.001; BV: OR, 0.24 (95% CI, 0.15–0.38), *P* < 0.001).

### Mediation analysis

Structured equation modeling and mediation analysis were used to investigate the direct effects of RoR and SES on pregnancy outcome, and the indirect effects when mediated through GA at diagnosis (Table 2, Figure 2). Using linear regression modeling, RoR was associated with an increase of 0.37 weeks in GA at diagnosis per 100‐km increment (β = 0.37; *P* = 0.015) and higher SES was associated with a reduction of 0.65 weeks in GA at diagnosis per unit on the Chan SES index scale (β = –0.65; *P* = 0.036). GA at diagnosis was associated with a 15% lower RR of TOP per week of gestation (RR, 0.85 (95% CI, 0.83–0.87); *P* < 0.001).

**Table 2:** Mediation analysis of indirect and direct effects of remoteness of residence (RoR) from a tertiary fetal cardiology center and Chan socioeconomic status (SES) index on rate of termination of pregnancy in cases with prenatal diagnosis of major congenital heart disease

Variable	Indirect effect	*P*	Direct effect	*P*	RIT	RID
RoR (/100km)	–0.061 ± 0.026	0.022	–0.017 ± 0.056	0.76	0.78	3.52
SES (Chan scale)	0.106 ± 0.050	0.053	0.177 ± 0.130	0.17	0.37	0.60

Indirect and direct effects are given as beta coefficient mean ± standard error. Ratio of indirect‐to‐total effect (RIT) and ratio of indirect‐to‐direct effect (RID) values quantify the proportions of the effects mediated by gestational age at diagnosis.

RoR ≥ 100 km was associated with a 6% reduction in TOP per 100‐km increment through an indirect effect via later GA at diagnosis (RR, 0.94 (95% CI, 0.89–0.99); *P* = 0.022), with 78% of the total effect mediated (RIT, 0.78). A RID of 3.52 for RoR estimates that the indirect effect was 3.5 times stronger than the direct effect. A trend towards an association between higher SES and increased likelihood of TOP, mediated by earlier GA at diagnosis, was also demonstrated (RR, 1.11 (95% CI, 1.00–1.22); *P* = 0.053). For SES, the RIT of 0.37 indicates that 37% of the total effect was mediated by GA at diagnosis, with a RID of 0.60 showing that the direct effect was stronger than the indirect effect (Table 2). These findings emphasize that, while the predictors (RoR and SES) have opposing effects, both operate through the common mediator of GA at diagnosis.

In pregnancies complicated by a syndromic diagnosis, there was no direct effect of RoR or SES on the rate of TOP, but a lower Chan SES index score was indirectly associated with a lower rate of TOP, mediated by GA at diagnosis (*P* = 0.02), and greater RoR showed a trend toward a similar effect (*P* = 0.057) (Table 3). Similar results were obtained for Chan SES index quintiles (Figure S1). There was no direct or indirect effect of RoR or SES on TOP in pregnancies without a syndromic diagnosis (Figure S2).

**Table 3:** Mediation analysis of indirect and direct effects of remoteness of residence (RoR) from a tertiary fetal cardiology center and Chan socioeconomic status (SES) index on rate of termination of pregnancy in cases with prenatal diagnosis of major congenital heart disease (mCHD), stratified by presence of comorbid syndromic diagnosis

Variable	Indirect effect	*P*	Direct effect	*P*	RIT	RID
Syndromic mCHD (*n* = 250)						
RoR (/100km)	–0.086 ± 0.045	0.057	–0.009 ± 0.088	0.92	0.90	9.56
SES (Chan scale)	0.241 ± 0.100	0.02	0.029 ± 0.215	0.89	1.14	NA*
Non‐syndromic mCHD (*n* = 771)						
RoR (/100km)	–0.059 ± 0.032	0.061	–0.034 ± 0.073	0.68	0.66	1.95
SES (Chan scale)	0.070 ± 0.059	0.29	0.273 ± 0.160	0.093	0.20	0.26

Data are given as mean ± standard error. Ratio of indirect‐to‐total effect (RIT) and ratio of indirect‐to‐direct effect (RID) values quantify the proportions of the effects mediated by gestational age at diagnosis. Owing to incomplete data, 250/268 syndromic cases and 771/823 non‐syndromic cases were included in mediation analysis.

* Cannot calculate ratio owing to difference in coefficient trend signs. NA, not applicable.

Stratified mediation analysis in BV physiology cases with complete data (*n* = 762) resembled the findings in the total cohort, with lower SES and greater RoR associated with later GA at diagnosis and with reduced RR of TOP via an indirect effect (Figure S3). In SV cases with complete data (*n* = 253), SES and RoR were not associated with later GA at diagnosis or a reduced rate of TOP (Figure S4).

### Sensitivity analysis

Mediation analysis provided a robust model for predictive measures of pregnancy outcome and GA at diagnosis. Using linear regression modeling and categorical RoR and SES measures, RoR ≥ 100 km was associated with an additional 1.72 weeks of GA at diagnosis (β = 1.72; *P* < 0.001), and higher Chan SES index quintile was associated with a reduction of 0.27 weeks of GA at diagnosis per quintile (β = –0.27; *P* = 0.026) (Table S4).

Analysis using a Poisson surrogate for the log‐binomial analysis prediction of pregnancy outcome demonstrated that the rate of TOP was reduced by 15% for every week taken to diagnose mCHD (RR, 0.85 (95% CI, 0.83–0.87); *P* < 0.001), with no significant direct effects of Chan SES index quintile, RoR ≥ 100 km or syndromic diagnosis on TOP. There was a 4% indirect effect and a 10% total effect on TOP per Chan SES index quintile (indirect: RR, 1.04 (95% CI, 1.01–1.08), *P* = 0.023; total effect: RR, 1.10 (95% CI, 1.00–1.20), *P* = 0.043). There was a –24% indirect effect and a −27% total effect of RoR ≥ 100 km on TOP (indirect effect: RR, 0.76 (95% CI, 0.66–0.88), *P* < 0.001; total effect: RR, 0.73 (95% CI, 0.52–1.10), *P* = 0.063). Similar results were obtained by logit analysis (Table S4).

Using causal mediation methodology to investigate the NIE, a 5% increase in the rate of TOP was associated with each quintile increase in Chan SES index (quintile 5 *vs* quintile 1: RR, 1.19 (95% CI, 1.04–1.36); *P* = 0.011), with no significant natural direct effect. RoR ≥ 100 km was associated with a NIE of a 22% reduction in the rate of TOP (RR, 0.78 (95% CI, 0.68–0.90); *P* = 0.001) (Table S5).

### Exploratory analysis

In addition to the main model, we explored the timing of diagnosis and rate of TOP in subgroups stratified by maternal age and presence of a comorbid syndromic diagnosis. Overall, no consistent differences in the rate of TOP were observed among RoR or SES categories when stratified by maternal age (< 35 years and ≥ 35 years) and by presence of a comorbid syndromic diagnosis. However, in a small subgroup of women < 35 years with a comorbid syndromic diagnosis, those residing ≥ 100 km from a tertiary fetal cardiology center were more likely to continue the pregnancy (*P* = 0.042) (Table S3). In contrast, RoR ≥ 100 km and lower SES category were associated with diagnosis ≥ 22 weeks (*P* < 0.001) when maternal age was < 35 years, but not when it was ≥ 35 years, irrespective of the presence or absence of a comorbid syndromic diagnosis (Table S3). Further subdivision of maternal age and consideration of parity demonstrated that the rate of TOP differed by maternal age category in parous mothers (*P* = 0.002), but not in nulliparous mothers (*P* = 0.81). Those with maternal age ≥ 45 years were less likely to continue the pregnancy (*P* = 0.014), and more likely to have a timely diagnosis (*P* = 0.005). This association was found irrespective of parity status, although the sample size for this subgroup was small (Table S2). There was a significantly lower prevalence of syndromic diagnoses among younger mothers (< 35 years; 167/809 (20.6%)) compared with that in older age groups (≥ 35 years; 101/282 (35.8%)) (*P* < 0.001).

## DISCUSSION

### Main findings and interpretation

Our study, encompassing 1091 pregnancies with a prenatal diagnosis of mCHD in Alberta, Canada, and known pregnancy outcome, provides insights into the interplay between maternal RoR, SES, age and obstetric history, and the impact of these variables on shaping pregnancy choice in the case of mCHD. Timely prenatal diagnosis of mCHD (< 22 weeks' gestation) was associated with a higher rate of TOP (27.9%) compared with a diagnosis later in pregnancy (8.8%), emphasizing the pivotal role of early detection in parental choice of pregnancy continuation, where available. No direct associations between TOP and maternal age, parity, RoR, SES or syndromic diagnosis were observed in the analysis; however, greater RoR was associated indirectly with a lower rate of TOP via later GA at diagnosis, and there was a trend towards a similar finding for lower SES.

While increased remoteness from a tertiary fetal cardiology center and lower SES were associated with a later GA at diagnosis of mCHD in the overall cohort, this effect was particularly evident in women < 35 years of age, with no significant association observed in older age groups. Our findings suggest that younger mothers who reside in remote areas, particularly those with a comorbid syndromic diagnosis of mCHD, may be unfairly disadvantaged by challenges in accessing healthcare, leading to later diagnosis and a consequent impact on the choice of pregnancy continuation. Access to timely antenatal screening via primary care and obstetric and radiological services should be improved to reduce geographical disparities in prenatal healthcare services. In pregnancies that are continued, early diagnosis of mCHD allows for better preparation and planning, allows more time for additional testing, facilitates access to specialized care and enables timely intervention, which can significantly impact the outcome for both the mother and the fetus^22^, ^23^, ^24^. Younger women residing in remote areas may miss the advantages of an earlier diagnosis, as our study found that later GA at diagnosis was more common among younger mothers living remotely. This may reflect differences in healthcare‐seeking behavior, social support or access to prenatal care among younger women, which can impact on timely referral and diagnosis.

An important finding of this study is the lack of a direct association between SES quintile and the rate of TOP. The temptation to assume that differences in the rate of TOP according to SES are due to education or career aspirations is not borne out by these results. Rather, vulnerability to a later diagnosis may predominantly underlie these findings. In a stratified analysis of cases with SV physiology, no association was identified between RoR or SES with GA at diagnosis or rate of TOP. These findings suggest that, while delayed diagnosis plays a key mediating role in pregnancy decision‐making for all mCHD, the severity of the mCHD may dominate decision‐making in more severe cases. However, lower power to detect an effect in a small SV physiology cohort may have contributed to this finding, particularly as fewer cases with SV physiology were diagnosed ≥ 22 weeks.

Previous research has highlighted an association between lower SES and delayed initiation of prenatal care^25^, ^26^, ^27^, ^28^, and lower overall prenatal detection rates are particularly significant in vulnerable populations^6^, ^7^, ^13^, ^25^, ^29^. These results reinforce the need for equitable access to early fetal cardiac assessment and comprehensive counseling.

The mediating role of the timing of prenatal diagnosis in this study underscores the need for targeted interventions to enhance accessibility, particularly in populations with potentially limited access to resources or in remote populations. For those with a pregnancy complicated by mCHD, contributing factors to delayed diagnosis may include limited social support, cultural and language barriers, lack of transportation, lack of financial support or employment security, and inadequate access to healthcare facilities^15^, ^30^, ^31^, ^32^, ^33^, ^34^. It is unclear at which point our cohort experienced delays prior to fetal cardiology diagnosis, such as when seeking primary care, obstetric screening and referral, access to a fetal cardiology center or other barriers. Further investigations are underway by our group to identify modifiable factors related to RoR and SES. Conversely, more prominent advantages in social capital and support in mothers ≥ 35 years of age may explain the higher proportion of timely diagnosis in this age group. Exploration is necessary to examine how social capital and community support can compensate for individual disadvantage, which can be exacerbated during pregnancy. Improving comprehensive prenatal care for vulnerable populations is crucial to ensure equitable and timely diagnosis of mCHD.

### Strengths

We chose to perform mediation analysis *a priori* as this method allows for the exploration of both direct and indirect relationships between SES, RoR and TOP, with GA at diagnosis serving as a potential mediating factor. This methodology is particularly suitable for understanding complex pathways where intermediate variables (such as GA at diagnosis) may play a significant role in influencing the outcome. Mediation analysis can detect indirect effects even when the direct association between the predictor and the outcome is not significant. In terms of causal interpretation of the demonstrated associations, our data benefit from a clear temporal relationship, with RoR and SES evidently present and important at the time of fetal diagnosis. Sensitivity analyses utilizing alternative mediation modeling parameters produced robust estimates of effects.

### Limitations

In the present study and in a previous study^11^, we utilized a cut‐off of < 22 weeks' gestation to define timely diagnosis. The differences in the rate of TOP seen in this study attest to the significance of this GA in influencing parental decision‐making. Although there is no gestation beyond which TOP is illegal in Alberta, Canada, we speculate that the requirement for ethical review board approval beyond 23 + 6 weeks is a significant consideration for most parents.

Furthermore, given that genetic testing using chromosomal microarray often requires 10–14 days, a diagnosis ≥ 22 weeks allows insufficient time to obtain these important results. Nevertheless, differences in legislation and cultural perspectives may invalidate this gestational cut‐off point in some healthcare services. We acknowledge that mCHD complexity, an important determinant of pregnancy outcome, was not included in the model owing to lack of data on standardized complexity scoring. Additionally, while maternal age was stratified to explore its influence on the rate of TOP, future studies incorporating age as a covariate in mediation models could provide further insight.

Finally, our study is subject to the usual limitations regarding statistical power to detect differences between groups, limiting our ability to examine some subpopulations. This study is also subject to the usual limitations regarding retrospective design, with the potential for selection bias and incomplete data. The existence of a single pediatric surgical center and close collaboration between the only two fetal cardiac services in Alberta, Canada, serve to minimize the risk of omitted cases; however, we cannot exclude the existence of rare unknown and unreferred cases of mCHD resulting in IUFD, or cases of TOP without a diagnosis or those prior to referral to fetal cardiology. This potential bias may result in underestimating the total number of cases of TOP. Geographic and socioeconomic factors can vary across different regions and healthcare systems, and thus our findings in Alberta may not be transferable to all jurisdictions in Canada or other universal healthcare systems. We acknowledge that religious beliefs and cultural backgrounds, which are important determinants of decisions about pregnancy, were not directly included in our model. The Chan SES index was used as a proxy for cultural influences and has been found to perform better than other Canadian SES indices for this component of cultural identity^20^. Future studies including individual‐level data on religion and culture could strengthen the understanding of these relationships. This study focused on the association between RoR, neighborhood‐level SES and pregnancy continuation choice, and did not explore the underlying mechanisms or patient‐specific factors contributing to the observed associations. Further research is needed to elucidate the complex interplay of these factors.

### Conclusions

In this retrospective cohort of prenatally diagnosed cases of mCHD, RoR from a tertiary fetal cardiology center and SES were associated with differences in pregnancy choice regarding TOP or continuation of pregnancy, mediated through later GA at prenatal diagnosis. The effects of maternal RoR were most prominent in mothers < 35 years of age, in whom SES also had an effect, and in pregnancies complicated by a syndromic diagnosis. Understanding these complexities is crucial to address the inequities in prenatal care and support parental choice in pregnancies facing the challenges associated with mCHD.

## Supporting information


**Table S1** Characteristics of study subjects according to timing of diagnosis.
**Table S2** Associations between maternal age categories and parity, as well as maternal age in the presence or absence of comorbid syndromic diagnosis, with gestational age at diagnosis and pregnancy outcome.
**Table S3** Associations between remoteness of residence (RoR) and socioeconomic status (SES) on pregnancy outcome and timing of prenatal diagnosis, stratified by maternal age.
**Table S4** Mediation analysis using alternative methods.
**Table S5** Causal mediation model showing effect of remoteness of residence ≥ 100km from closest tertiary fetal cardiology center on pregnancy outcome, directly and when mediated by gestational age at diagnosis (as a continuous variable).
**Figures S1 and S2** Relationship between socioeconomic status (SES), remoteness of residence (RoR) and pregnancy outcome mediated by gestational age (GA) at prenatal diagnosis, in patients with comorbid syndromic diagnosis (Figure S1) and those without comorbid syndromic diagnosis (Figure S2).
**Figures S3 and S4** Relationship between socioeconomic status (SES), remoteness of residence (RoR) and pregnancy outcome mediated by gestational age (GA) at prenatal diagnosis, in patients with biventricular physiology (Figure S3) and those with single ventricular physiology (Figure S4).

## Data Availability

The data that support the findings of this study are available on request from the corresponding author. The data are not publicly available due to privacy or ethical restrictions.
