# Supplementary material for: Impact of socioeconomic status and remoteness of residence on pregnancy outcome in major congenital heart disease: mediation analysis
Source: Ultrasound Obstet Gynecol. 2026 Jan 3;68(1):51–8. doi: 10.1002/uog.70145 (PMC13325687; doi:10.1002/uog.70145)
Supplement: Supplementary file 1 — Table S1 Characteristics of study subjects according to timing of diagnosis. Table S2 Associations between maternal age categories and parity, as well as maternal age in the presence or absence of comorbid syndromic diagnosis, with gestational age at diagnosis and pregnancy outcome. Table S3 Associations between remoteness of residence (RoR) and socioeconomic status (SES) on pregnancy outcome and timing of prenatal diagnosis, stratified by maternal age. Table S4 Mediation analysis using alternative methods. Table S5 Causal mediation model showing effect of remoteness of residence ≥ 100km from closest tertiary fetal cardiology center on pregnancy outcome, directly and when mediated by gestational age at diagnosis (as a continuous variable). Figures S1 and S2 Relationship between socioeconomic status (SES), remoteness of residence (RoR) and pregnancy outcome mediated by gestational age (GA) at prenatal diagnosis, in patients with comorbid syndromic diagnosis (Figure S1) and those without comorbid syndromic diagnosis (Figure S2). Figures S3 and S4 Relationship between socioeconomic status (SES), remoteness of residence (RoR) and pregnancy outcome mediated by gestational age (GA) at prenatal diagnosis, in patients with biventricular physiology (Figure S3) and those with single ventricular physiology (Figure S4). [file UOG-68-51-s001.docx]

**Supplemental Tables & Figures**

**Table S1: Characteristics of study subjects according to timing of diagnosis**

|  | **GADx<23 weeks** |  | **GADx** $\mathbf{<}$ **24 weeks** |  |
| --- | --- | --- | --- | --- |
|  | *n/N (%)* | *p-value* | *n/N (%)* | *p-value* |
| **Total** | 671/1098  (61.1%) |  | 740 /1098 (67.4%) |  |
| **Distance from fetal centre**  **<100 km**  **≥100 km** | 540/841 (64.2%)  131/257 (51.0%) | <0.0001 | 593/841 (70.5)  147/257 (57.2) | <0.0001 |
| **Chan Index Quintile**  **1**  **2**  **3**  **4**  **5** | 124/222 (55.9)  111/206 (53.9)  147/222 (66.2)  117/197 (59.4)  137/194 (70.6) | 0.002 | 145/222 (65.3)  127/206 (61.7)  157/222 (70.6)  129/197 (65.5)  142/194 (73.2) | 0.095 |
| **Maternal Age, years -Categorical**  <35  35-39  40-44  ≥45 | 449/823 (60.6)  133/200 (66.5)  33/65 (50.8)  6/10 (60.0) | 0.14 | 547/823 (66.5)  147/200 (73.5)  40/65 (61.5)  6/10 (60.0) | 0.17 |
| **Maternal parity**  **0**  **≥1** | 269/440 (61.1)  400/656 (61.0) | 0.96 | 296/440 (67.3)  442/656 (67.4) | 0.97 |
| **Syndromic diagnosis** |  |  |  |  |
| **No** | 501/830 (60.4) | 0.37 | 547/830 (65.9) | 0.06 |
| **Yes** | 170/268 (63.4) |  | 193/268 (72.0) |  |

Legend: Subjects were divided into two groups, timely diagnosis group as those with prenatal diagnosis < 23weeks’, or <24 weeks’ gestation, and late diagnosis as those diagnosed at and after 23 weeks’ or 24 weeks’ gestation. Abbreviations: confidence interval (CI); gestational age at diagnosis of major congenital heart disease (GADx); socioeconomic status (SES). ^†^ Chi-squared result for testing statistical significance.

Table S2: Associations between maternal age categories and parity, as well as maternal age in the presence or absence of comorbid syndromic diagnosis, with gestational age at diagnosis (GADx) and pregnancy outcomes.

|  | **Continuation of pregnancy** |  | **GADx**  $\mathbf{<}$ **22 weeks** |  |
| --- | --- | --- | --- | --- |
|  | *n/N (%)* | *p-value* | *n/N (%)* | *p-value* |
| **Maternal age (in years) & nulliparous**  **<35**  **35-39**  **40-44**  **>45** | 289/357 (81.0)  48/58 (82.8)  14/16 (87.5)  2/2 (100.0) | 0.809 | 190/359 (52.9)  34/58 (58.6)  3/16 (18.8)  2/2 (100.0) | 0.018 |
| **Maternal age (in years) & parity ≥1**  **<35**  **35-39**  **40-44**  **>45** | 378/452 (83.6)  108/139 (77.7)  40/49 (81.6)  9/18 (50.0) | 0.002 | 218/455 (47.9)  79/141 (56.0)  23/49 (46.9)  14/18 (77.8) | 0.036 |
| **Maternal Age (in years) & No Syndromic diagnosis**  **<35**  **35-39**  **40-44**  **>45** | 531/642 (82.7)  109/134 (81.3)  29/34 (85.3)  7/13 (53.8) | 0.057 | 329/647 (50.8)  72/136 (52.9)  11/34 (32.34)  11/13 (84.6) | 0.013 |
| **Maternal Age (in years) & Syndromic diagnosis**  **<35**  **35-39**  **40-44**  **>45** | 136/167 (81.4)  47/63 (74.6)  25/31 (80.6)  4/7 (57.1) | 0.333 | 79/167 (47.3)  41/63 (65.1)  15/31 (48.4)  5/7 (71.4) | 0.072 |

The Pearson Chi-square was significant (p<0.001) and a demonstrate significantly lower prevalence of syndromic diagnosis in younger mothers (less than 35 years) and higher prevalence of syndromic diagnoses in mothers aged 35-44 years. There was no significant difference between mothers 45 years and older compared to those under 45 years of age. Logistic regression was used to determine odds ratio and 95% CIs.

Table S3: Association between remoteness of residence (ROR) and socioeconomic status (SES) on pregnancy outcome and timing of prenatal diagnosis, stratified by maternal age

|  | **Continuation of pregnancy** |  | **GADx**  $\mathbf{<}$ **22 weeks** |  |
| --- | --- | --- | --- | --- |
|  | *n/N (%)* | *p-value* | *n/N (%)* | *p-value* |
| **Remoteness** |  |  |  |  |
| **Maternal age <35years**  **<100 km**  **≥100 km**  **≥35 years**  **<100 km**  **≥100 km** | 378/453 (83.4)  140/158 (88.6)  148/181 (81.8)  17/22 (77.3) | 0.12  0.61 | 251/453 (55.4)  50/158 (31.6)  97/181 (53.6)  12/22 (54.6) | <0.001  0.932 |
| **Socioeconomic status** | |  |  |  |
| **Maternal age <35years**  **1 (lowest)**  **2**  **3**  **4**  **5 (highest)**  **≥35 years**  **1 (lowest)**  **2**  **3**  **4**  **5 (highest)** | 139/162 (85.8)  128/154 (83.1)  127/156 (81.4)  125/148 (84.5)  116/149 (77.8)  52/59 (88.1)  39/50 (78.0)  48/64 (75.0)  36/49 (73.5)  33/44 (75.0) | 0.402  0.318 | 76/163 (46.6)  58/155 (37.4)  91/158 (57.6)  71/148 (48.0)  89/150 (59.3)  27/59 (45.8)  30/51 (58.8)  35/64 (54.7)  26/49 (53.1)  27/44 (61.4) | 0.001  0.546 |
| **Without comorbid syndromic diagnosis** | | | | |
| **Maternal age <35 years & ROR**  **<100 km**  **≥100 km**  **≥35 years**  **& ROR**  **<100 km**  **≥100 km** | 312/368 (84.8)  103/118 (87.3)  98/120 (81.7)  9/11(81.2) | 0.503  0.99 | 210/368 (57.1)  39/118 (33.0)  65/120 (54.2)  5/11 (54.6) | <0.001  0.579 |
| **Maternal age <35 & SES**  **1 (lowest)**  **2**  **3**  **4**  **5 (highest)**  **≥35 years**  **& SES**  **1 (lowest)**  **2**  **3**  **4**  **5 (highest)** | 117/133 (88.0)  94/115 (81.7)  99/122 (81.2)  102/119 (85.7)  93/120 (77.5)  32/34 (94.1)  28/33 (84.8)  30/43 (69.8)  22/29 (75.9)  22/29 (75.9) | 0.205  0.089 | 63/134 (47.0)  48/116 (41.4)  75/124 (60.5)  58/119 (48.7)  68/121 (56.2)  16/34 (47.1)  18/34 (52.94)  24/43 (55.8)  12/29 (41.4)  17/29 (58.6) | 0.025  0.664 |
| **With comorbid syndromic diagnosis** | | | | |
| **Maternal age <35 & ROR**  **<100 km**  **≥100 km**  **≥35 years**  **& ROR**  **<100 km**  **≥100 km** | 66/85 (77.6)  37/40 (92.5)  50/61 (82.0)  8/11 (72.7) | 0.042  0.476 | 41/85 (48.2)  44/85 (51.8)  32/61 (52.5)  7/11 (63.6) | 0.028  0.493 |
| **Maternal age <35 & SES**  **1 (lowest)**  **2**  **3**  **4**  **5 (highest)**  **≥35 years**  **& SES**  **1 (lowest)**  **2**  **3**  **4**  **5 (highest)** | 22/29 (75.9)  34/39 (87.2)  28/34 (82.4)  23/29 (79.3)  23/29 (79.3)  20/25 (80.0)  11/17 (64.7)  18/21 (85.7)  14/20 (70.0)  11/15 (73.3) | 0.805  0.576 | 13/29 (44.8)  10/39 (25.6)  16/34 (47.1)  13/29 (44.8)  21/29 (72.4)  11/25 (44.0)  12/17 (70.6)  11/21 (52.4)  14/20 (70.0)  10/15 (66.7) | 0.005  0.285 |

The Pearson Chi2 was significant (p<0.001) and a demonstrate significantly less diagnosis prior to 22 weeks gestation in younger mothers in remote and lower SES categories. This was consistent in pregnancies with and without comorbid syndromic diagnosis. There was significance association between continuation of pregnancy in mothers younger than 35 who were remote with a pregnancy complicated by comorbid syndromic diagnosis (p=0.042). Abbreviations: gestational age at diagnosis (GADx); remoteness of residence (ROR); termination of pregnancy (TOP); socioeconomic status (SES).

**Table S4 –** Mediation analysis using alternative methods

| GSEM, Poisson regression, log link, robust VCE: Predictor – Mediator - Outcome | | | | | | | |
| --- | --- | --- | --- | --- | --- | --- | --- |
|  | Predictors | RR - direct (95% CI) | p-value | RR-indirect (95% CI) | p-value | RR - Total effects (95% CI) | p-value |
| ToP | GADx (weeks) | 0.85 (0.83, 0.87) | <0.001 |  |  |  |  |
|  | Chan Index quintile | 1.05 (0.97, 1.15) | 0.24 | 1.04 (1.01, 1.08) | 0.023 | 1.10 (1.00, 1.20) | 0.043 |
|  | Distance (≥100km) | 0.96 (0.70, 1.30) | 0.78 | 0.76 (0.66, 0.88) | <0.001 | 0.73 (0.52, 1.101) | 0.063 |
|  | Syndrome (Y/N) | 1.08 (0.82, 1.4) | 0.59 | 1.14 (1.01, 1.29) | 0.037 | 1.23 (0.92, 1.64) | 0.17 |
|  |  |  |  |  |  |  |  |
| GSEM, logit model: Predictor – Mediator - Outcome | | | | | | | |
|  |  | Odds ratio  (direct) (95% CI) |  | Odds ratio  (indirect)  (95% CI) |  | Odds ratio  Total effect  (95% CI) |  |
| ToP | GADx (weeks) | 0.81 (0.77, 0.85) | <0.001 |  |  |  |  |
|  | Chan Index quintile | 1.08 (0.0.95, 1.21) | 0.23 | 1.06 (1.01, 1.12) | 0.031 | 1.14 (1.00, 1.30) | 0.046 |
|  | Distance (≥100km) | 0.97 (0.63, 1.47) | 0.89 | 0.69 (0.57, 0.83) | <0.001 | 0.67 (0.42, 1.05) | 0.082 |
|  | Syndrome (Y/N) | 1.12 (0.77, 1.64) | 0.55 | 1.20 (1.00, 1.42) | 0.045 | 1.34 (0.89, 2.02 | 0.16 |
| Linear model: Predictor - Mediator | | | | | | | |
|  |  | Coefficient (95% CI) |  |  |  |  |  |
| GADx | Chan Index quintile | -0.27 (-0.51, -0.03) | 0.026 |  |  |  |  |
|  | Distance (≥100km) | 1.72 (0.93, 2.51) | <0.001 |  |  |  |  |
|  | Syndrome (Y/N) | -0.83 (-1.60, -0.04) | 0.039 |  |  |  |  |

**Table S5**: Causal mediation model showing effect of remoteness of residence ≥100km from closest fetal cardiology center on pregnancy outcome, directly and when mediated by gestation at diagnosis (as a continuous variable).

| **Distance Category (**≥**100km)** |  | **RR** | **p-value** |
| --- | --- | --- | --- |
| **NIE** |  | 0.78 (0.68, 0.90) | 0.001 |
|  |  |  |  |
| **NDE** |  | 0.94 (0.70, 1.28) | 0.72 |
|  |  |  |  |
| **TE** |  | 0.74 (0.53, 1.03) | 0.07 |
|  |  |  |  |
| **Chan Index Quintile (vs 1)** |  |  |  |
| **NIE** | 2 | 1.05 (1.01 ,1.09) | 0.013 |
|  | 3 | 1.10 (1.02, 1.18) | 0.01 |
|  | 4 | 1.15 (1.02, 1.27) | 0.01 |
|  | 5 | 1.19 (1.04, 1.36) | 0.011 |
|  |  |  |  |
| **NDE** | 2 | 1.06 (0.96, 1.16) | 0.25 |
|  | 3 | 1.11 (0.93, 1.34) | 0.25 |
|  | 4 | 1.17 (0.90, 1.54) | 0.25 |
|  | 5 | 1.24 (0.86, 1.77) | 0.24 |
|  |  |  |  |
| **TE** | 2 | 1.11 (1.00,1.22) | 0.047 |
|  | 3 | 1.22 (1.01,1.48) | 0.043 |
|  | 4 | 1.34 (1.01,1.78) | 0.039 |
|  | 5 | 1.47 (1.03,2.12) | 0.036 |

Syndromic/genetic diagnosis (Y/N) was included as a covariate. Inclusion of maternal age (as a continuous variable) and/or parity (as a count) did not materially change the model predictions and reduced overall fit, therefore were excluded for simplicity. Outcome (ToP) was modelled as using logit, and gestational age at diagnosis in weeks was modelled using Poisson. NIE: Natural Indirect Effect, NDE: Natural Direct Effect, TE: Total Effect

Figure S1. Relationship between socioeconomic status (SES), remoteness of residence (RoR) and pregnancy outcome mediated by gestational age (GA) at prenatal diagnosis, in patients with comorbid syndromic diagnosis.
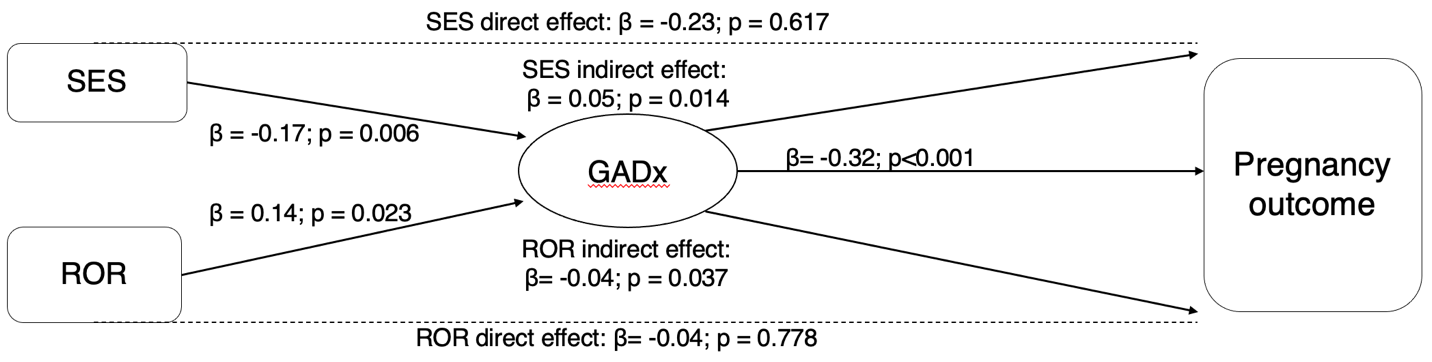


Dashed lines represent associations that are not statistically significant and solid lines represent associations that are significant. Later GADx has a significant direct effect with lower likelihood of termination of pregnancy (p<0.001). The SES (Chan index quintiles) and RoR (<100 km ≥100 km) variables on the left are seen to have no significant association with the pregnancy outcome directly (p=0.617 and p=0.778 respectively). In syndromic patients, the effect of lower SES is significantly associated with delayed GADx (p=0.006) and has a significant effect on pregnancy outcome when mediated via GADx (p=0.014). Greater ROR is associated with later GADx (p=0.023) and there is a lower likelihood of termination of pregnancy when ROR is mediated through GADx (p=0.037). Beta is the coefficient in linear model.

Figure S2. Relationship between socioeconomic status (SES), remoteness of residence (RoR) and pregnancy outcome mediated by gestational age (GA) at prenatal diagnosis, in patients without syndromic diagnosis.
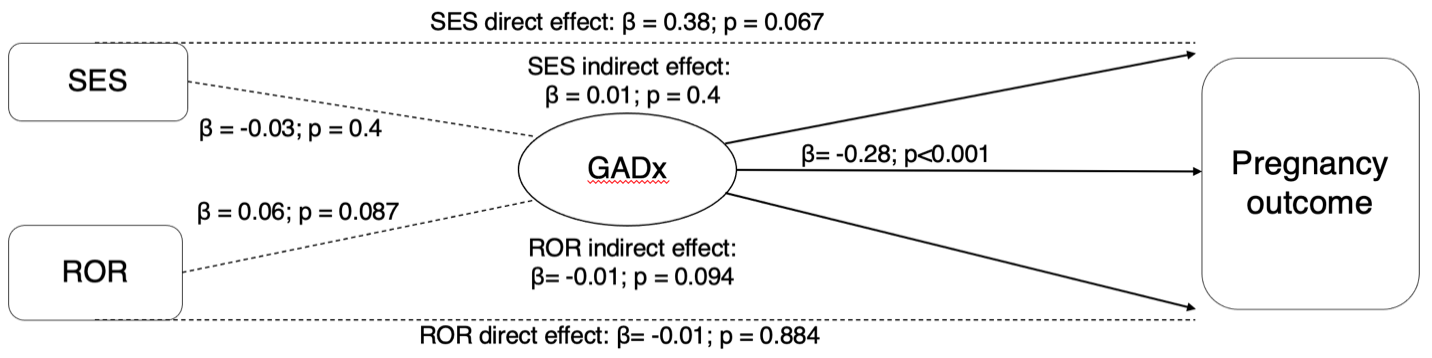


Dashed lines represent associations that are not statistically significant and solid lines represent associations that are significant. Later GADx has a significant direct effect with lower likelihood of termination of pregnancy (p<0.001). In patients without a comorbid diagnosis, SES (Chan index quintiles) and RoR (<100 km ≥100 km) variables on the left are seen to have no significant direct effect (p=0.067 and p=0.884 respectively) or indirect effect (p=0.4 and p=0.094 respectively) with the pregnancy outcome. Beta is the coefficient in linear model.

Figure S3. Relationship between socioeconomic status (SES), remoteness of residence (RoR) and pregnancy outcome mediated by gestational age (GA) at prenatal diagnosis, in patients with biventricular physiology.


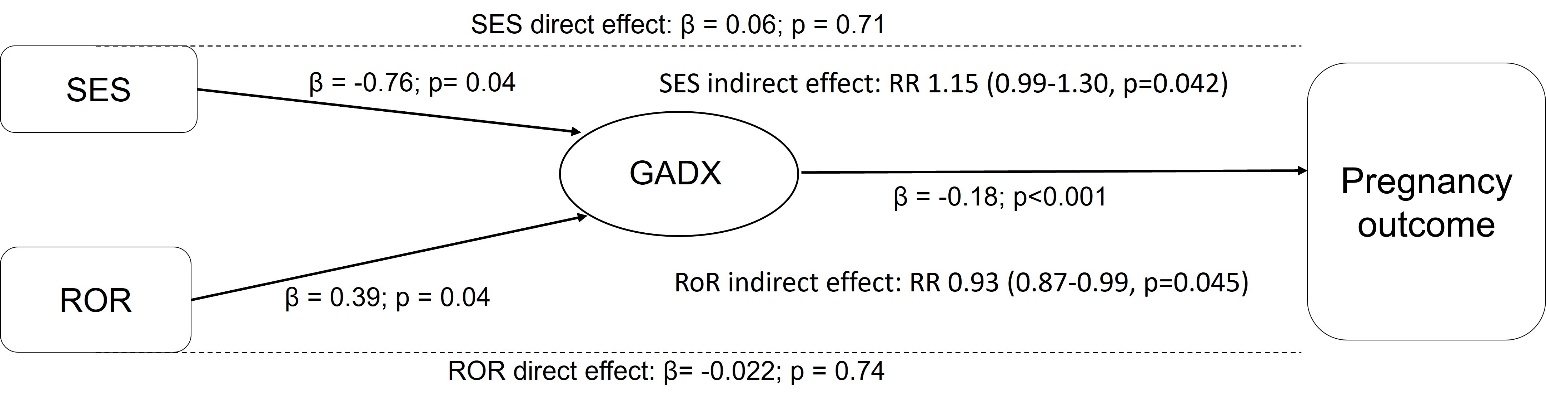


Dashed lines represent associations that are not statistically significant and solid lines represent associations that are significant. Later GADx has a significant direct effect with lower likelihood of termination of pregnancy (p<0.001). In patients with biventricular physiology, SES (Chan index quintiles) and RoR (per 100 km) variables on the left are seen to have no significant direct effect but do have a significant indirect relationship with pregnancy outcome. Beta is the coefficient in linear model.

Figure S4. Relationship between socioeconomic status (SES), remoteness of residence (RoR) and pregnancy outcome mediated by gestational age (GA) at prenatal diagnosis, in patients with single ventricular physiology.
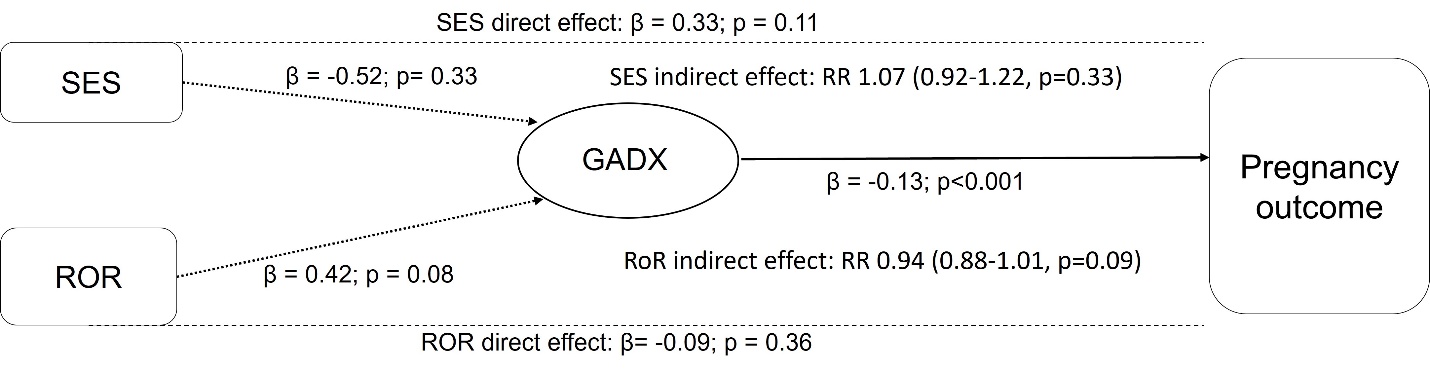


Dashed lines represent associations that are not statistically significant and solid lines represent associations that are significant. Later GADx has a significant direct effect with lower likelihood of termination of pregnancy (p<0.001). In patients with single ventricular physiology, neither SES (Chan index quintiles) or RoR (per 100 km) have a significant direct effect or indirect effect on pregnancy outcome. Beta is the coefficient in linear model.
